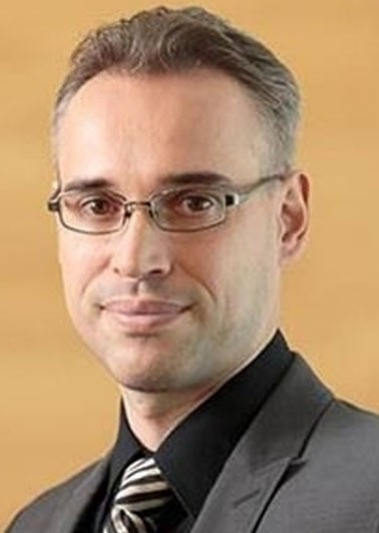# Functional Redox‐Active Molecular Tunnel Junctions

**DOI:** 10.1002/asia.202000932

**Published:** 2020-10-14

**Authors:** Yingmei Han, Christian A. Nijhuis

**Affiliations:** ^1^ Department of Chemistry National University of Singapore 3 Science Drive 3 Singapore 117543 Singapore; ^2^ Centre for Advanced 2D Materials and Graphene Research Centre National University of Singapore 6 Science Drive 2 Singapore 117546 Singapore

**Keywords:** molecular electronics, redox-active molecules, molecular switches, charge transport, tunnelling

## Abstract

Redox‐active molecular junctions have attracted considerable attention because redox‐active molecules provide accessible energy levels enabling electronic function at the molecular length scales, such as, rectification, conductance switching, or molecular transistors. Unlike charge transfer in wet electrochemical environments, it is still challenging to understand how redox‐processes proceed in solid‐state molecular junctions which lack counterions and solvent molecules to stabilize the charge on the molecules. In this minireview, we first introduce molecular junctions based on redox‐active molecules and discuss their properties from both a chemistry and nanoelectronics point of view, and then discuss briefly the mechanisms of charge transport in solid‐state redox‐junctions followed by examples where redox‐molecules generate new electronic function. We conclude with challenges that need to be addressed and interesting future directions from a chemical engineering and molecular design perspectives.

## Graphical abstract

**In recent years, redox‐active molecules** have contributed significantly to the development of functional molecular junctions including molecular diodes, molecular memory and molecular transistors. This minireview gives an overview of recent progresses in functional redox‐active molecular junctions and introduces the underlying operating mechanisms of the different types of functional molecular junctions.

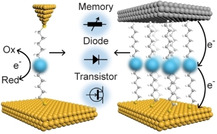

## 1 Introduction

Molecular electronics is complementary to conventional semiconductor based electronics with the ultimate goal of generating ultra‐small, molecular‐scale electronic devices whose properties can be controlled via atomistic engineering by changing the chemical structure of the molecules.^[1]^ Despite impressive demonstrations of molecular control over electronic function, such as, molecular rectification,[^1c^, ^2^] molecular memory,^[3]^ molecular transistors,^[4]^ or negative differential resistance,^[5]^ it is still challenging to rationally design junctions because the molecule interacts with at least two electrodes forming a new physical‐organic system. This means that, besides the chemical structure of the molecule, it is equally important to control the nature of the molecule‐electrode interfaces,^[6]^ energy level alignment of the molecular energy levels with respect to the Fermi‐levels of the electrodes,^[7]^ and the supramolecular structure of the junction.^[8]^ Thus, molecular electronics is a highly interdisciplinary field bridging chemistry, physics, engineering, and theory.

**Figure 1:**
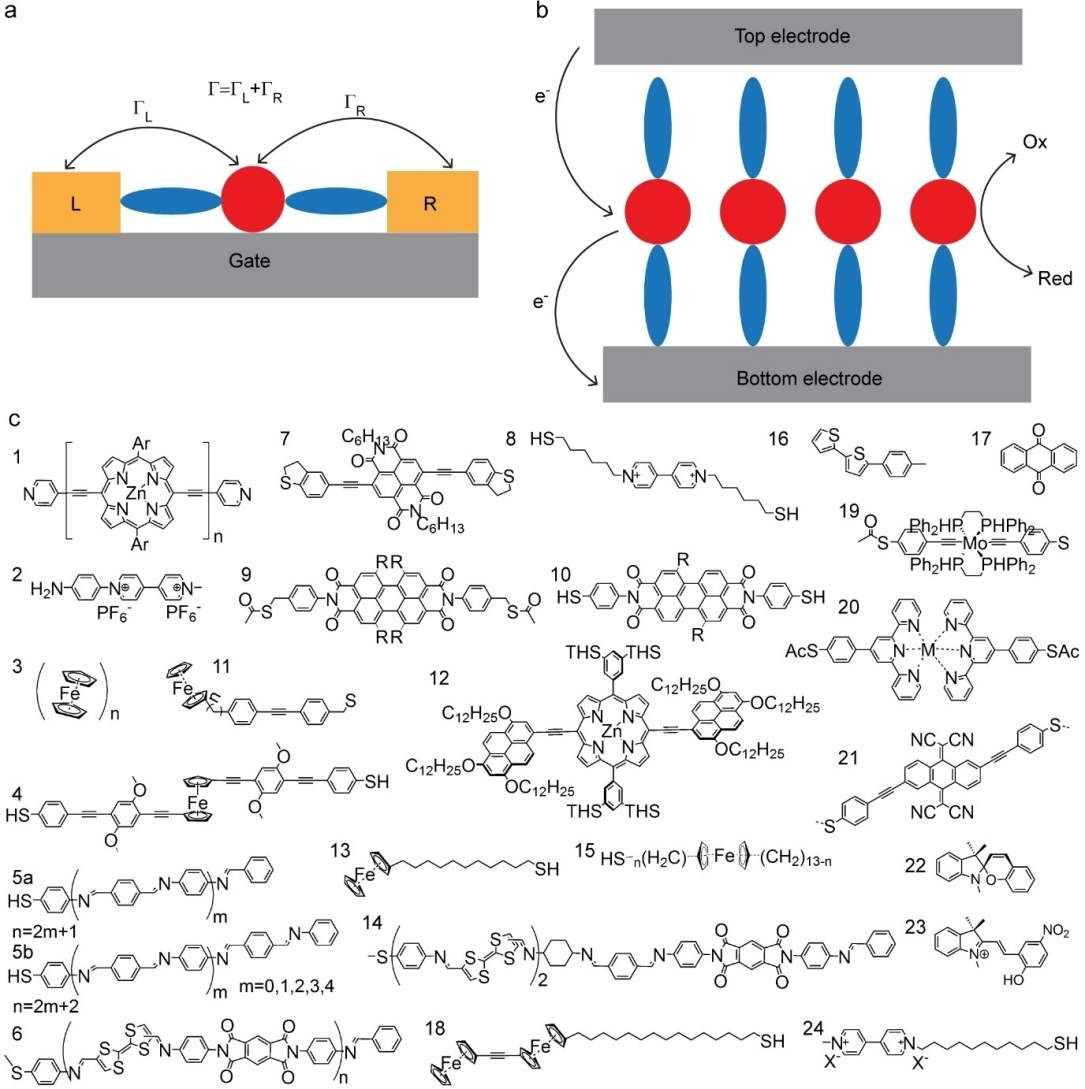
Schematic illustrations of a single‐molecule junction (a) and large‐area molecular junction (b). The red spheres represent the redox‐units and the blue ovals represent the molecular backbone. L and R denote left electrode and right electrode. (c) Chemical structures of redox‐molecules discussed in this Review where n denotes the number of repeat units, R denotes tert‐butyl‐phenoxy, pyrrolidinyl, chlorine, and thiobutyl moieties, Ph=phenyl, Ar=aryl, X^−^=counter ion, Ac=acetyl and THS=trihexylsilyl.

Molecular junctions can be categorized into single‐molecule junctions (Figure 1a) where one molecule bridges two electrodes, and large‐area junctions (Figure 1b) where a monolayer of molecules separates the two electrodes.^[1g]^ The advantage of large‐area junctions is that the monolayer structure can be characterized in great detail before the top‐contact is introduced. In contrast, single‐molecule junctions are made *in situ* by repetitive making and breaking of the junctions where molecules are trapped by chance from dilute solutions. Consequently, there is virtually no information available on the chemical and supramolecular organization of single‐molecule junctions. Although single‐molecule junctions are assumed to be “single‐molecule”, neighboring molecules are present (in other words, single‐molecule junctions consist of disordered and dilute monolayers), which are usually ignored. Thus, it is important to realize that in analysis of data obtained from single‐molecule junctions usually a “perfect” junction structure is assumed without experimental verification.

Incorporating redox‐active molecules in molecular junctions (Figure 1c) is an attractive approach to generate functional molecular junctions because redox‐molecules have molecular frontier orbitals that are close in energy to the Fermi‐levels of the electrode materials that are typically used (*e. g*., Au, Ag, or Pt).^[9]^ These low‐lying molecular energy levels can readily participate in the mechanism of charge transport, and due to the redox‐nature of such molecules, the charge carriers are expected to interact strongly with the molecules. Such strong interactions between charge carriers and redox‐molecules in molecular junctions have been evidenced by *in situ* optical methods (albeit it with ill‐defined junctions) where excitation of molecular vibration modes,^[10]^ light emission,^[11]^ and junction dynamics^[12]^ could be monitored while measuring the current‐voltage response of the junctions. Whether in‐ or coherent tunnelling dominates the mechanism of charge transport depends not only on the redox‐properties of the molecule, but also on the molecule‐electrode coupling strength (Γ) which determines by how much the molecular frontier orbitals are broadened and follow the changes in the Fermi energy of the electrode, *E*_F_, under applied voltage bias, *V*. In the weak coupling limit, the charge carriers can interact strongly with molecules resulting in formal redox‐reactions inside the junctions and the mechanism of charge transport is incoherent tunnelling (which is also called hopping). In the strong coupling limit, the charge carriers interact weakly with the molecules. Consequently, redox‐reactions cannot occur and coherent tunnelling dominates the mechanism of charge transport. Although different redox‐states of molecules are readily stabilized under wet‐electrochemical conditions by solvent molecules and counterions, these stabilization mechanisms are not present in solid‐state molecular junctions, and, instead, charges on the molecule induce image charges in the electrodes.[^1c^, ^1g^, ^13^] These image charge effects alter the energy level alignment of the junction and associated tunnelling barrier heights, and, in turn, the measured currents and tunnelling decay coefficient *β* (low *β* values are associated with good molecular conductors while high *β* values are associated with insulators). To fully appreciate the differences between redox‐processes under wet‐electrochemical conditions and in solid‐state junctions, we introduce briefly the single‐level Landauer theory for coherent tunnelling,^[14]^ and the Marcus theory of hopping,^[15]^ and how both theories are applied to described charge transport in solid‐state molecular junctions.

Recently, reviews have appeared which give a broad overview of the field of molecular electronics, the type of electronic functions that can be achieved, and how molecular junctions are fabricated.[^1a^, ^1b^, ^1c^, ^1d^, ^1e^, ^1f^] This minireview aims to introduce the concept of “redox‐active junctions” from a molecular design perspective, how redox‐molecules affect the mechanism of charge transport in solid‐state tunnel junctions and generate electronic functionalities. We conclude the review with challenges that are needed to overcome and how this can be achieved from a molecular‐design approach.

## 2 Mechanisms of charge transport

Broadly speaking, the mechanisms of charge transport can be categorized as coherent and incoherent tunnelling which are described by the Landauer and Marcus theory, respectively, both of which rely on a number of assumptions. In reality, however, molecular junctions often show more complex behavior which cannot be fully understood within the framework of either theory and therefore a large number of extensions, alternatives, and combined theories have been proposed.[^5b^, ^9^, ^16^] A large number of reviews have been already dedicated to this topic,[^1g^, ^16i^, ^17^] in this Section we only briefly discuss the major concepts to define redox‐junctions.

### 2.1 Electronic structure of molecular junctions: definitions

**Figure 2:**
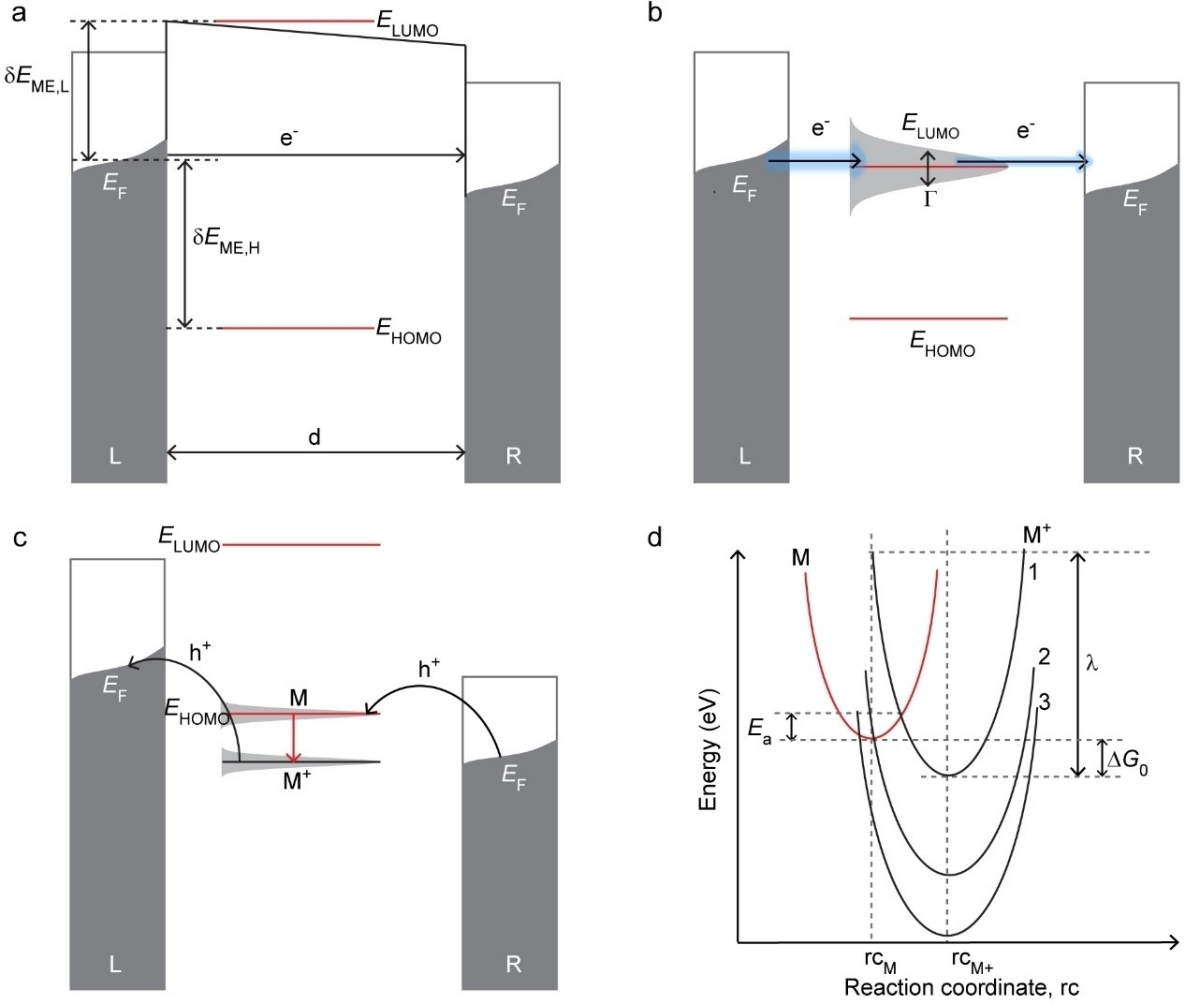
Energy level diagrams for coherent off‐resonant tunnelling (a), coherent resonant tunnelling (b) and incoherent tunnelling where M denotes a neutral, and M^+^ a charged, molecule (c). (d) Marcus parabolas for charge transfer from M to M^+^ in the normal Marcus (curve 1) and inverted Marcus regions (curve 3).

Figure 2 shows the energy level diagram of a molecular junction where the energy of the highest occupied (*E*_HOMO_), or lowest unoccupied molecular orbitals (*E*_LUMO_), with respect to *E*_F_ defines the tunnelling barrier height (δ*E*_ME,H_ or δ*E*_ME,L_), and the molecular length defines the tunnelling barrier width *d*. In case the molecular frontier orbitals fall outside the conduction window (δ*E*_ME,H_ and δ*E*_ME,L_> applied bias *V*), the mechanism of charge transport is off‐resonant coherent tunnelling (Figure 2a). However, in case the LUMO or HOMO falls inside the conduction window (δ*E*_ME,H_ or δ*E*_ME,L_ < *V*), resonant tunnelling dominates where the charge carriers can tunnel through this level, which increases the measured currents considerably. In this case, depending on how the charge carrier interacts with the molecules, the mechanism of charge transport can be coherent or incoherent tunelling.^[16e]^ In the case of strong molecule‐electrode coupling (given by Γ), the molecular orbital is broad and the coherent tunnelling rates are high (Figure 2b). When the molecule‐electrode coupling is weak, the charge carriers can relax on the molecule resulting in incoherent tunnelling (Figure 2c). In this case, the charge carrier interacts strongly with the molecule and therefore can couple to vibrational modes resulting in oxidation (or hole injection in the HOMO) or reduction (or electron injection in the LUMO). The Marcus theory is widely used to describe the electron transfer rate *k*_ET_ of incoherent charge transfer processes usually encountered under wet‐electrochemical conditions where the redox‐molecules undergo formal oxidation or reduction which are accompanied by structural reorganization within the molecule and solvent molecules given by re‐organization energy *λ*.^[15]^ Figure 2d shows the Marcus parabolas for a typical exothermic redox‐process where a neutral molecule M is oxidized to M^+^. Although Marcus theory usually describes solution based redox‐processes, below we discuss examples in solid‐state junctions.

### 2.2 Coherent off‐resonant tunnelling

Off‐resonant tunnelling is usually described by the Simmons theory[^1c^, ^1g^, ^17d^, ^18^] which was originally developed to describe the tunnelling current, *I*, through metal‐oxide barriers. In the low bias regime, *I* is given by:^[18c]^

(1) $$I=\frac{\alpha e^{2}}{4\pi^{2}\hbar^{2}d^{2}}{\left(2m_{e}{\delta E}_{ME}\right)}^{1/2}Vexp\left[-\frac{2\alpha d}{\hbar }{\left(2m_{e}{\delta E}_{ME}\right)}^{1/2}\right]$$

where *e* is the electron charge, *m*_e_ is the effective mass of the electron, *α* is a parameter to modify the tunnelling barrier shape (to account, for instance, for image charge effects), and *ħ* is reduced Planck's constant. In this framework, the charge carrier tunnels across a potential barrier with the tunnel barrier height δ*E*_ME,H_ or δ*E*_ME,L_ and width *d* defined by the molecules. The role of image charge effects (which causes rounding, and lowering, of the tunnelling barrier height) and the meaning of effective electron mass in the context of molecular tunnel junctions are both unclear. Thus, a major shortcoming of this analysis is that image charge effects, effective electron mass, and the tunnelling barrier heights are usually treated as fitting parameters. Assuming linear potential drops, Equation 1 can be simplified to the “general tunnel equation”[^1c^, ^1g^, ^17d^, ^18b^, ^18c^]

(2) $$J=J_{0}e^{-\beta d}\;\mathrm{with}\;\beta =2\sqrt{\frac{2m_{e}\delta E_{ME}}{\hbar^{2}}}\;$$

which defines the tunnelling decay coefficient β , and *J*_0_ is a pre‐exponential factor (which is often seen as an effective contact resistance,[^18c^, ^19^] see ref 20 for more details). The value of *β* describes how well a molecule conducts and can be determined by measuring the exponential decay of the current with molecular length *d*. For instance, typically alkyl chains are considered to be insulating and have *β* values of 0.9–1.1 Å^−1^, while molecules with aromatic or conjugated backbones have low *β* values of 0.2–0.4 Å^−1^.[^1a^, ^1d^, ^1e^, ^1g^, ^18c^, ^19b^, ^19c^, ^21^] It is important to note that this mechanism of charge transport is independent of temperature *T*, apart from a weak temperature dependency associated with thermal broadening of the Fermi levels (see next Section).

### 2.3 Coherent resonant tunnelling

Tunnelling through molecular orbitals can be described by the Landauer‐Büttiker formalism[^1c^, ^1d^, ^1g^, ^16e^, ^17a^, ^17d^, ^22^] where the current depends on the transmission probability *T* of a charge carrier from one macroscopic electrode to another separated by a mesoscopic quantum mechanical scatter (here the molecule), and is given by

(3) $$I\left(V\right)=\frac{2e}{h}\int_{-\infty }^{+\infty }T\left(E,V\right)\left[f\left(E-\frac{eV}{2}\right)-f\left(E+\frac{eV}{2}\right)\right]dE$$

where *E* is the energy of the charge carrier, *f*(E) is the Fermi‐Dirac distribution of the electrodes given by Equation 4 and *T*(*E*,*V*) is the transmission function given by Equation 5

(4) $$f\left(E\right)=\frac{1}{\left[1+exp\left(\left(E-E_{F}\right)/k_{B}T\right)\right]}$$

(5) $$T\left(E,V\right)=\frac{4\Gamma_{L}\Gamma_{R}}{{\left[E-\delta E_{ME}\left(V\right)\right]}^{2}+{\left[\Gamma_{L}+\Gamma_{R}\right]}^{2}}$$

where *T* is absolute temperature, and *k*_B_ is the Boltzmann's constant, Γ_L_ and Γ_R_ denote the coupling with the left and right electrodes (Figure 1a), δ*E*_ME_ (*V*) is the energy offset between molecular energy level and *E*_F_ under applied bias *V*, which is given by Equation 6:

(6) $${\delta E}_{ME}\left(V\right)={\delta E}_{ME}+\left(\frac{\Gamma_{L}-\Gamma_{R}}{\Gamma_{L}+\Gamma_{R}}\right)\frac{eV}{2}\;$$

This framework accounts, unlike the Simmons equation, for molecule‐electrode interactions and associated broadening of molecular energy levels. Also, in this framework, charge transport is nearly independent of the applied temperature, but a thermal broadening of the Fermi‐levels can cause a small temperature dependency.^[16e]^ Finally, the transmission probability essentially determines *β* and also decreases exponentially with molecular length (*i. e*., the measured current also decreases with *d*, Equation 2) but with significantly smaller values of *β* (<0.1 Å^−1^)^[23]^ compared to that of coherent off‐resonant tunnelling (Section 2.2 and 3.1).

### 2.4 Incoherent tunnelling

Figure 2d shows the typical Marcus parabolas for a one‐step electron transfer M⇌M^+^ in solution where the Gibbs free energy of the reaction, Δ*G*_0_, is defined (curve 1). For an exothermic reaction with an energetically favourable electron transfer reaction where M^+^ has a lower energy than M (curve 1 in Figure 2d), the transition from M to M^+^ does not occur spontaneously because of the energy barrier between the two states. Thus, unlike in a coherent tunnelling process, the charge has to “hop” over the activation barrier (*E*_a_) defined by the intersecting parabolas. Assuming the Frank‐Condon principle holds,[^1c^, ^15^, ^16i^, ^17b^, ^17e^] electrons can only hop if the nuclei of M have a similar configuration as the nuclei of M^+^. This distortion of M is the transition state and requires energy. The Gibbs free energy of the formation of the transition state involves the re‐organization energy *λ* which is also defined in Figure 2d. In this framework, *k*_ET_ is given by^[1c]^

(7) $$k_{ET}=\left(\frac{2\pi }{\hbar }\right){\left|H_{MM^{+}}\right|}^{2}{\left(4\pi \lambda k_{B}T\right)}^{-\frac{1}{2}}\;exp\left[-\frac{{\left(\Delta G_{0}+\lambda \right)}^{2}}{4\lambda k_{B}T}\right]\;$$

where *H*_MM+_ is the electronic coupling strength between M and M^+^. This equation has the same form as the Arrhenius equation (note *J* is used instead of *k*_ET_ for straightforward comparison with Equation 2)

(8) $$J=J_{0}e^{-\frac{E_{a}}{k_{B}T}}\;\mathrm{with}\;E_{a}=\frac{{\left(\lambda +\Delta G_{0}\right)}^{2}}{4\lambda }\;$$

but, importantly, the Marcus theory provides a quantitative description of the activation energy *E*_a_ and the pre‐exponential factor *J*_0_.

Normally, incoherent tunnelling involves redox‐processes that are thermally activated while coherent tunnelling is essentially independent of *T*. Consequently, temperature‐dependent charge transport measurements are normally used to discriminate between the two processes. Charge transfer rates (or currents) dominated by coherent off‐resonant processes are very sensitive to the molecular length (*i. e*., large *β* values)[^1a^, ^1d^, ^1e^, ^1g^, ^18b^, ^18c^, ^21^] while junctions dominated by incoherent tunnelling depend weakly on the molecular length (*i. e*., very low *β* values).^[24]^ In practice, however, it turns out to be challenging to discriminate between in‐ and coherent tunnelling. For instance, charge transfer rates dominated by coherent resonant tunnelling may also be insensitive to the molecular length (*i. e*., small *β* values).[^23^, ^25^] On the other hand, activationless charge transport process does not necessarily indicate coherent tunnelling (Sections 2.5 and 3.4). For instance, Figure 2d also shows the situation (curve 2) where the Marcus parabolas intersect each other at the minimum resulting in *E*_a_=0 eV yet the charge transfer process is incoherent. Thermal broadening of the Fermi level (Equation 4) also results in a small thermally activated component in case of coherent tunnelling which could be mistakenly attributed to hopping. In case Δ*G*_0_ increases further, the charge transfer reaction slows down again due to the presence of an activation barrier (curve 3). This decrease in the electron transfer rate with increasing Δ*G*_0_ is called the inverted Marcus region. Although a reduction of *k*_ET_ with increasing Δ*G*_0_ has been observed in solution,^[26]^ in junctions the energy from the leads can compensate for *E*_a_ in the inverted region, resulting in activationless charge transport[^16b^, ^27^] (Sections 2.5 and 3.4).

### 2.5 Theories Beyond Landauer and Marcus

**Figure 3:**
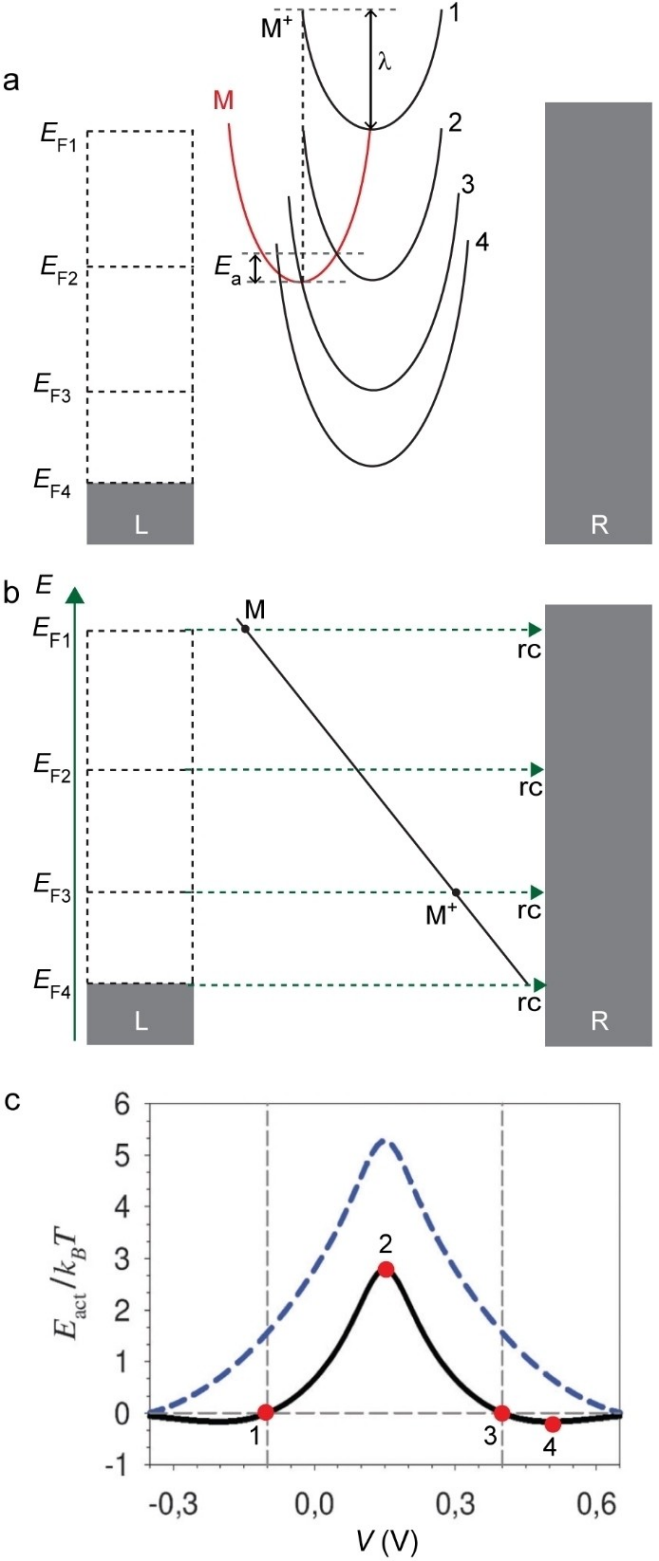
(a) Schematic illustration of a redox‐active junction between M and M^+^ where four different situations are indicated with the redox‐reaction occurring within the bias window, or outside the bias window. *E*_F1_ to *E*_F4_ denote the change of Fermi level under applied bias. (b) The corresponding redox‐reaction from M to M^+^ in relation to rc with the changing of *E*_F_. (c) The calculated *E*_a_ *vs. V* with *λ*=0.25 eV (black solid line) and *λ*=0.50 eV (blue dashed line). Reprinted with permission from ref 16b, copyright 2012 The Royal Society of Chemistry.

Coherent (Eqs. 1–6) and incoherent tunnelling (Eqs. 7 and 8) represent the two extremes to describe the mechanism of the charge transport. As mentioned earlier, molecular tunnel junctions, on the other hand, can operate in between these two regimes depending on the molecule‐electrode coupling strength, the availability of molecular energy levels and vibrational modes that resemble the transition of the redox reaction.[^16i^, ^28^] Although solid‐state junctions lack solvent molecules and counterions, the charge on the molecule can be readily stabilized by image charges in the electrodes due to the extremely large polarizability of metals.[^1c^, ^1g^, ^13^] To describe redox‐processes in junctions, various approaches have been reported.[^9^, ^16b^, ^16d^, ^16f^] For example, Migliore *et al*.^[16b]^ combined the Marcus and Landuaer theories to describe redox‐active tunnel junctions where the charge transfer rates between M and M^+^ are expressed as:

(9) $$k_{M\to M^{+}}=\frac{1}{\sqrt{4\pi k_{B}T}}\int dE\Gamma \left(E\right)e^{-\frac{{\left(\Delta E+E-\lambda \right)}^{2}}{4k_{B}T\lambda }}f\left(E\right)$$

(10) $$k_{M^{+}\to M}=\frac{1}{\sqrt{4\pi k_{B}T}}\int dE\Gamma \left(E\right)e^{-\frac{{\left(\Delta E+E+\lambda \right)}^{2}}{4k_{B}T\lambda }}\left(1-f\left(E\right)\right)\;$$

where *Γ*(*E*) is Fermi's golden rule charge transfer rate between the molecule energy level and electrode. Figure 3 shows the electrochemical reaction from M to M^+^ inside the junctions where it is important to note that points M and M^+^ represent different points on the reaction coordinate, rc, rather than a tunnelling distance. The energy difference (Δ*E*) between the energy of M (*E*_M_) and M^+^ (*E*_M+_) surface is given by $\Delta E=E_{M}\left({rc}_{M}\right)-E_{M^{+}}\left({rc}_{M^{+}}\right)+E_{F}$ where rc_M_ and rc_M+_ indicate the respective points on rc.

Migliore *et al*.^[16b]^ suggested that by applying a voltage to the electrodes, the redox reaction can be pushed into the inverted Marcus region (curve 4 in Figure 3a) as follows. Figure 3a shows this situation where Δ*G*_0_ changes due to changes of *E*_F_ under the action of applied *V* by, for instance, intramolecular orbital gating.^[27]^ Figure 3b shows the corresponding transition from M to M^+^ on the reaction coordinate. In the situation where *E*_F_ crosses M (curve 1 in Figure 3a and line *E*_F1_ in Figure 3b), the charge transport is activationless (point 1 in Figure 3c). With increasing bias, *E*_a_ reaches a maximum (point 2 in Figure 3c) when the minima of both the M and M^+^ parabolas have equal energy (curve 2 in Figure 3a and line *E*_F2_ in Figure 3b). Beyond this point *E*_a_ decreases again until *E*_a_=0 meV (point 3 in Figure 3c) when the parabolas intersect at their minima (curve 3 in Figure 3a and line *E*_F3_ in Figure 3b). Beyond this point, in principle the system enters the inverted Marcus region (curve 4 in Figure 3a and line *E*_F4_ in Figure 3b) and *E*_a_ increases again with *V*, but in this case the energy required to overcome *E*_a_ is available from the leads and charge transport across the junction is essentially activationless (point 4 in Figure 3c). Consequently, the *E*_a_ depends on *V* following a bell‐shaped curve (Figure 3c).

In single molecule experiments it is possible to identify the vibrational modes of the molecule that are excited during charge transport. Sowa *et al*.^[16d]^ followed a generalized quantum master equation approach to derive the charge transfer rates *k* of redox junctions given by

(11) $$k_{red}=2Re\left[\Gamma \int_{-\infty }^{\infty }\frac{dE}{2\pi }f\left(E\right)\int_{0}^{\infty }dt\;e^{+i\left(E-{\delta E}_{ME}\right)t}e^{-\Gamma t}B\left(t\right)\right]$$

(12) $$k_{ox}=2Re\left[\Gamma \int_{-\infty }^{\infty }\frac{dE}{2\pi }\left[1-f\left(E\right)\right]\int_{0}^{\infty }dt\;e^{-i\left(E-{\delta E}_{ME}\right)t}e^{-\Gamma t}B\left(t\right)\right]$$

where *B*(t) is the time dependent phononic correlation function, and the subscripts ox and red denote oxidation and reduction processes. The current is given in the following expression:

(13) $$I=e\frac{k_{red}^{L}k_{ox}^{R}-k_{red}^{R}k_{ox}^{L}}{k_{red}^{L}+k_{ox}^{R}+k_{red}^{R}+k_{ox}^{L}}\;$$

where the super scripts L and R denote the left and right electrode. This model also bridges the Marcus theory and the Landauer formalism, but this framework is especially useful to extract the vibrational reorganization energy (Section 3.4).

## 3 Examples of redox‐active junctions in different charge transport regimes

### 3.1 Coherent resonant tunnelling

**Figure 4:**
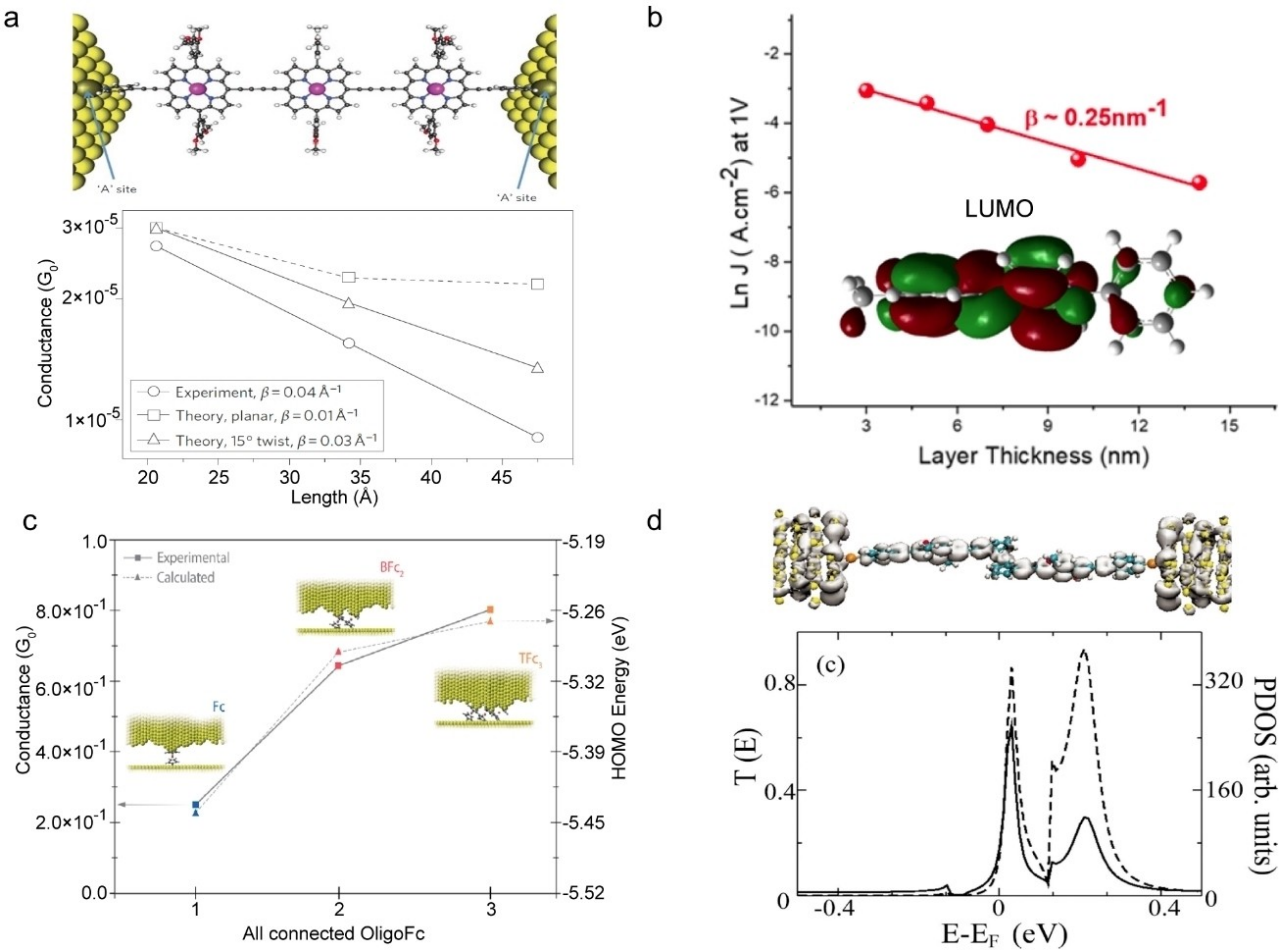
(a) Single‐molecule junction with compound **1** and the experimentally and theoretically obtained *G vs. d* plots. Reprinted with permission from ref 25a, copyright 2011 Nature Springer. (b) ln *J vs. d* plot at 1 V for large‐area junction with compound **2** and DFT calculated LUMO for compound **2**. Reprinted with permission from ref 25b, copyright 2018 American Chemical Society. (c) Value of *G* of single‐molecule junctions with (Fc)_n_ molecular wires with n=1, 2, or 3 (compound **3**). Reprinted with permission from ref 30, copyright 2019 American Chemical Society. (d) Local density of states (LDOS) at the peak of the transmission curve shown below of the single‐molecule junction with compound **4** with the transmission through the junction (dashed line) and projected density of states on the molecule (solid line). Reprinted with permission from ref 31, copyright 2005 American Physical Society.

As mentioned in Sections 2.2 and 2.4, normally the values of *β* for aliphatic or conjugated molecules are relatively high mainly due to large values of δ*E*_ME_. In contrast, redox‐active molecules have the *E*_HOMO_ or *E*_LUMO_ close in energy to *E*_F_ and, consequently, low δ*E*_ME_ and small *β*[^23a^, ^23b^] (Eqs. 1 and 2). As explained in Section 2.3, coherent tunnelling across redox‐molecules can dominate the mechanism of charge transport provided that *Γ* is large enough which is usually the case in single molecule junctions. Extremely low *β* values have been reported for junctions with redox‐active molecules.[^23^, ^25^, ^29^] For example, Sedghi *et al*.^[25a]^ reported *β*=0.04 Å^−1^ for oligo‐porphyrin wires compound **1** (Figure 1c) in single molecule junctions (Figure 4a) due to resonant tunnelling. Similarly, Nguyen *et al*.^[25b]^ also reported a low *β* value of 0.025 Å^−1^ (Figure 4b) across molecular wires with viologen repeating units (compound **2**). The energetically low‐lying LUMO of the viologen (which is −4.5 eV) falls in between the Fermi levels of the Au (−5.1 eV) and Ti (−4.1 eV) electrodes, along with a large Γ induced by the covalent Au−C bond, both point to a resonant tunnelling process across these molecular junctions.

The importance of coupling strength between molecular orbitals and the electrode in resonant tunnelling process is further demonstrated by Aragonès *et al*.^[30]^ who reported near ballistic (*T(E,V)* is close to 1, Equation 5) charge transport across ferrocene (Fc) oligomers compound **3** with n=1, 2 and 3 in scanning tunnelling microscope (STM) break junctions (Figure 4c). Density functional theory (DFT) showed that resonant tunnelling is mediated by the delocalized HOMO over the Au electrodes due to the high reactivity of the uncoordinated gold atoms of the break junction resulting in a large Γ. As a result, the junction has a large conductance *G*=0.8 *G*_0_ where *G*_0_=2*e* ^2^/*h* (the maximum conductance through a single channel with a transmission probability of 1). A similarly high conductance with *G*=0.7 *G*_0_ has also been observed by Getty *et al*.^[31]^ across Fc‐oligophenylethynyl dithiol (compound **4**) in single‐molecule junctions (Figure 4d). Figure 4d shows that molecular orbital that is responsible for conduction is delocalized over the entire molecule and the electrodes, and the DFT calculated transmission curves indicate the presence of resonance peak just 30 meV above *E*_F_ (Figure 4d) explaining the large value of *G*.

### 3.2 Transition from coherent to incoherent tunnelling

**Figure 5:**
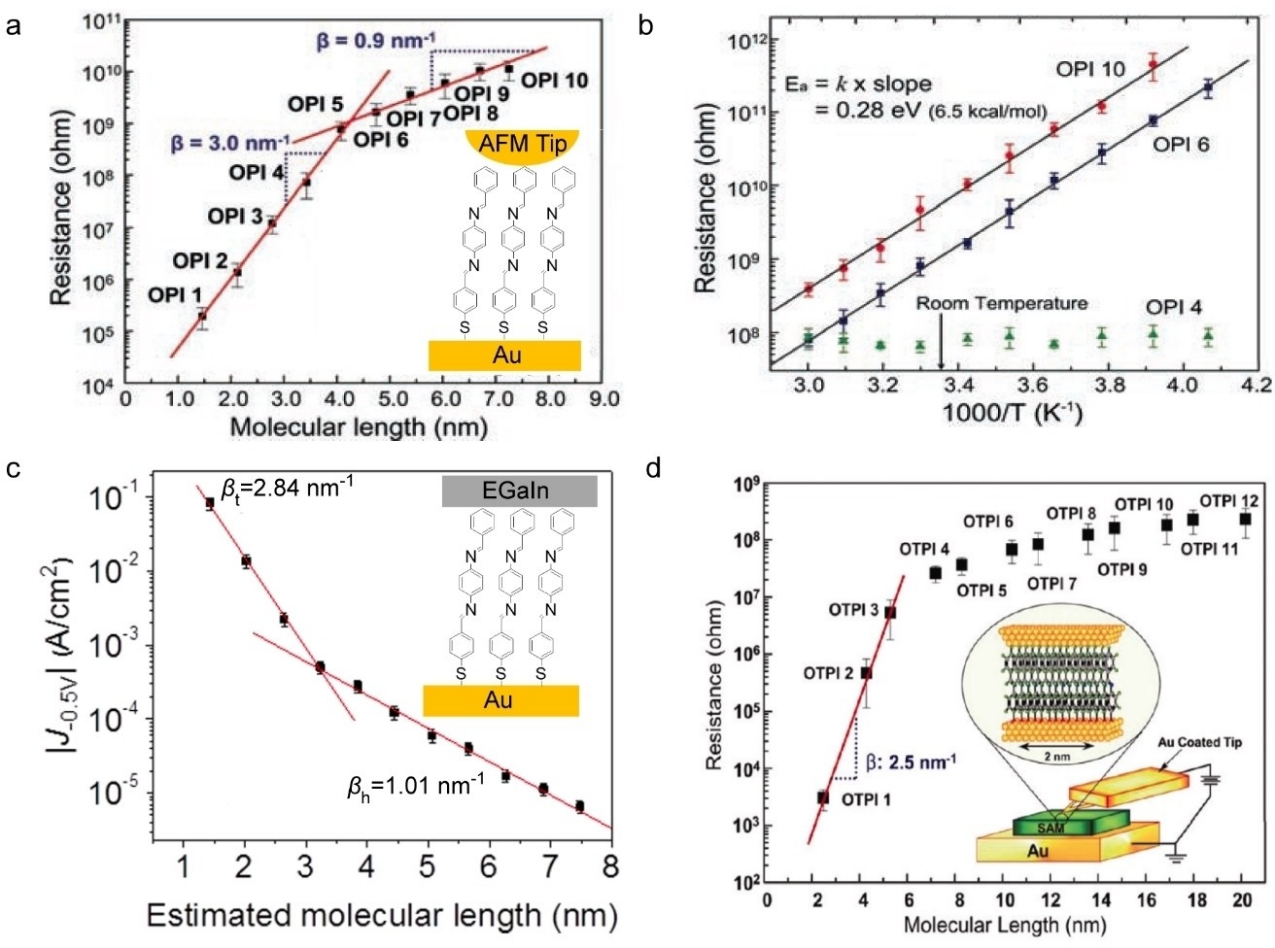
(a) Resistance *vs. d* for AFM based junctions with compound **5** shown in the inset and (b) corresponding Arrhenius plots for OPI 4, 6 and 10. Reprinted with permission from ref 32a, copyright 2008 AAAS. (c) *J vs. d* for large‐area junctions with OPI and schematic illustration of the junction. Reprinted with permission from ref 32b, copyright 2016 American Chemical Society. (d) Resistance *vs. d* for AFM based junctions with compound **6** and schematic illustration of the junction. Reprinted with permission from ref 32c, copyright 2010 American Chemical Society.

Coherent tunnelling always dominates the mechanism of charge transport for sufficiently small values of *d*, while incoherent tunnelling always dominates for sufficiently large *d*.[^17a^, ^24c^, ^32^] Therefore, for a given molecular wire a transition from coherent to incoherent tunnelling should exist as a function of *d*. The group of Frisbie^[32a]^ reported this transition for monolayers of oligophenyleneimine (OPI) molecular wires compound **5** in contact with atomic force microscopy (AFM) tip as the top electrode with varying length of 1.5 to 7.3 nm by changing the number of OPI repeat units n from 1 to 10. Figure 5a shows the resistance of the junction as a function of n where *β*=0.3 Å^−1^ is much larger for small values of n <5, than that for junctions with large values of n >5 with *β*=0.09 Å^−1^. Consequently, charge transport is activationless for n <5, and is thermally activated with *E*_a_=280 meV for n >5 (Figure 5b). Sangeeth *et al*.^[32b]^ demonstrated this transition in large‐area junctions with eutectic gallium indium alloy (EGaIn) as the top electrode (Figure 5c). In these large‐area junctions, *β* value is 0.28 Å^−1^ for n <5, and *β*=0.10 Å^−1^ for n >5 indicating that the same transition occurs as in the AFM‐based junctions. A similar transition has been reported for junctions with other types of molecular wires including tetrathiafulvalene (TTF) ‐ pyromellitic diimide (PMDI) oligoimine (OPTI, compound **6**) as shown in Figure 5d.^[32c]^ Yan *et al*.^[33]^ observed three mechanisms of charge transport with sharp transitions in 4.5 to 22 nm thick large‐area molecular junctions: coherent off‐resonant tunnelling for *d*<8 nm, activated hoping for *d*>16 nm and activationless field induced ionization for *d*=8 to 22 nm. In contrast, Chen *et al*.^[34]^ reported a gradual transition to incoherent tunnelling along monolayers consisting of peptides indicating partial loss of coherence during charge transport. These examples show that the various charge transport regimes can be accessed, but it is not well‐understood why transitions are abrupt or how much coherence is lost.

### 3.3 Charge transport in the Normal Marcus region

**Figure 6:**
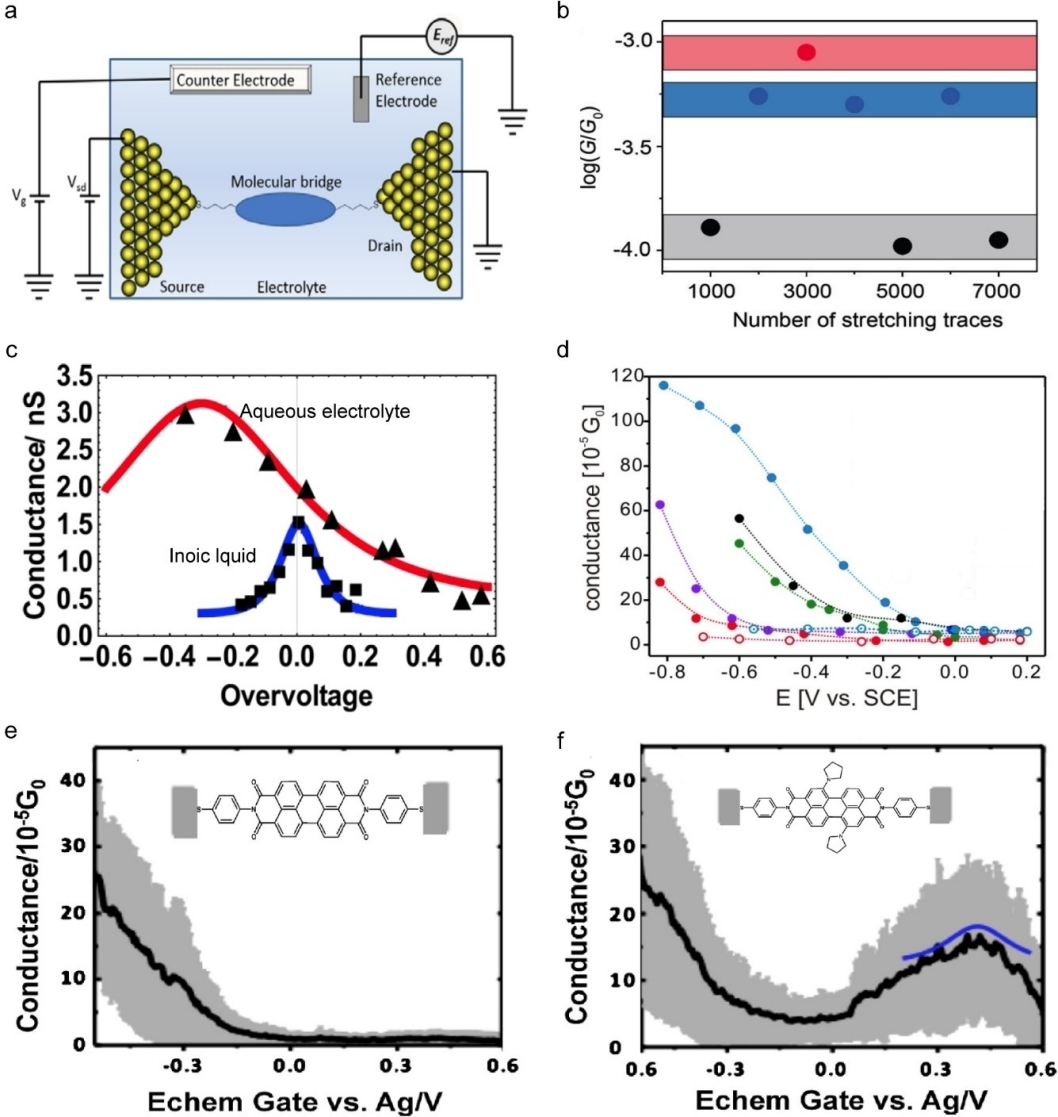
(a) Schematic illustration of an electrochemically gated single‐molecule junction. Reprinted with permission from ref 36, copyright 2016 American Chemical Society. (b) Single‐molecule conductance for neutral NDI (compound **7**, black), NDI radical anion (blue) and NDI dianion (red). Reprinted with permission from ref 37, copyright 2015 Wiley‐VCH. (c) Conductance *vs*. applied electrode potentials in aqueous (red) and ionic liquid electrolyte (blue) for single‐molecule junction with compound **8**. Reprinted with permission from ref 39, copyright 2015 American Chemical Society. (d) Single‐junction conductance *vs*. applied electrode potentials in junction with compound **9**. Reprinted with permission from ref 42, copyright 2013 Wiley‐VCH. Conductance *vs*. electrochemical gate voltage for single‐molecule junctions with the unsubstituted (e) and substituted (f) PTCDI (compound **10**). Reprinted with permission from ref 43, copyright 2012 American Chemical Society.

To control the redox‐state of a molecule inside a junction, a gate electrode is required (Figure 1a). Instead of a solid‐state gate electrode, a single‐molecule junction can also be immersed in an electrolyte solution where the junction can be gated via the electrolyte. Such electrochemical molecular junctions have been realized with, *e. g*., electrochemical STM based break junctions, electromigrated break junctions, or junctions with scanning electrochemical microscopes.^[35]^ Figure 6a shows the concept of electrochemically gated single‐molecule junctions where the four electrodes allow for independent potential control over the redox‐molecules with respect to the electrodes.^[36]^ By using this junction configuration, Li *et al*.^[37]^ have demonstrated conductance switching in a naphthalenediimide (NDI, compound **7**) based junction where NDI is an electron acceptor which can be reduced via two consecutive one‐electron steps. This change of the redox‐state from neutral NDI to the dianion results in a factor of 10 change in the conductance of the junction (Figure 6b). Similar increase of conductance upon oxidation has also been reported by Chen *et al*.^[38]^ Osorio *et al*.^[39]^ demonstrated the influence of electrolyte on the gating efficiency of a single molecule junctions with a viologen derivative **8**. Figure 6c shows that by using an ionic liquid 1‐butyl‐3‐methylimidazolium triflate (BMIM‐OTf) as the electrolyte, a sharp peak was observed in the conductance as a function of electrochemical gating voltage with the maximum centered at the formal redox potential of **8** ^2+^/**8** ^.+^ redox‐couple, and a gate coupling efficiency of 100 % was obtained by fitting the data with a two‐step hopping model proposed by Kuznetsov and Ulstrup.^[40]^ However, it is worth to note that non‐redox active molecular junctions can also be gated by the electrolyte to control the energy level alignment of the junction resulting in 3‐fold increase in the conductance.^[41]^ In addition, the molecular structures also influence the gating efficiency. For instance, Li *et al*.^[42]^ demonstrated that for symmetrical perylene tetracarboxylicbisimides (PBI, compound **9**) derivatives, a distinct gating effect on the conductance was observed which is related to the reduction ability of the molecule tuned by the substituents (Figure 6c, solid circle). While for asymmetric molecules, there is no significant change of the conductance with the electrochemical potential (Figure 6c, empty circle). Similar results have been observed by Díez‐Pérez *et al*.^[43]^ in perylenetetracarboxylic diimide (PTCDI, compound **10**) based single‐molecule junctions (Figure 6e and 6 f). On the other hand, the on/off ratio can be improved by optimizing the molecule‐electrode coupling strength. For instance, Li *et al*.^[44]^ demonstrated a three‐fold imporvement in the on/off ratio by changing the anchoring group from a thiol to a carbodithioate.

### 3.4 Charge transport in other tunnelling regions

**Figure 7:**
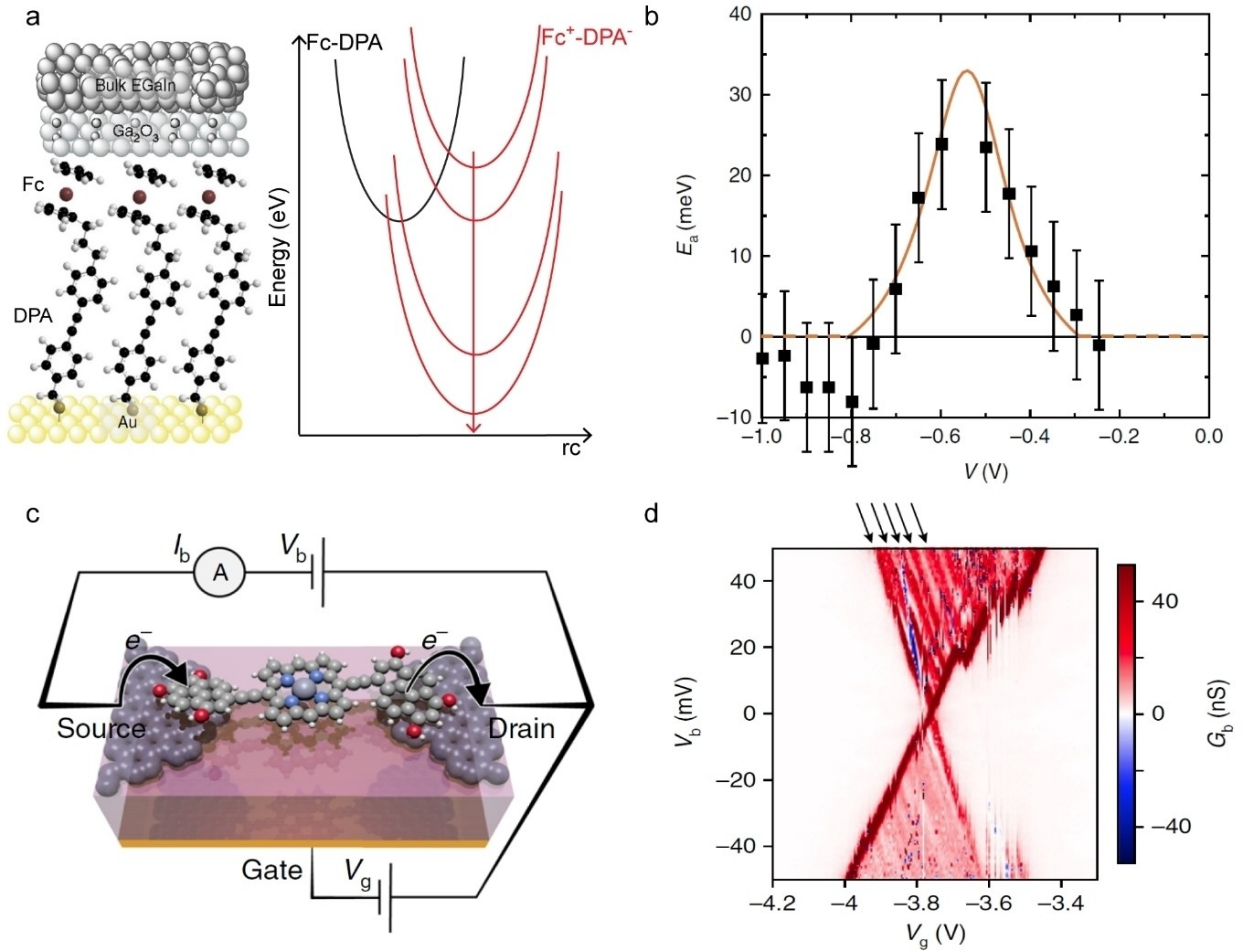
(a) Large‐area junction with compound **11** and corresponding Marcus parabolas and (b) the experimental *E*_a_ *vs. V* (symbols) with fit to Marcus‐Landauer theory (Eqs. 9 and 10; orange line). Reprinted with permission from ref 27, copyright 2018 Nature Springer. (c) Schematic representation of a single‐molecule junction with compound **12** and (d) the charge stability diagram of differential conductance (*G*_b_) as a function of gate voltage (*V*_g_) and bias voltage (*V*_b_) at 3.5 K. Arrows indicate vibrational excitations. Reprinted with permission from ref 45, copyright 2019 Nature Springer.

As mentioned in Section 2.5, redox junctions can be pushed into the inverted Marcus region resulting in activationless hopping providing the Marcus parabolas can be shifted with respect to each other (Figure 3). Figure 7a shows a molecular junction with donor‐bridge‐acceptor (D−b−A ) compound **11** with Fc as the donor and diphenylacetylene (DPA) as the acceptor where charge can hop from the donor to acceptor as indicated by the Marcus parabolas in Figure 7a.^[27]^ Charge can be readily injected into the Fc which results in “intramolecular orbital gating” (which was confirmed with single‐molecule measurements^[27]^) reducing the energy of the LUMO with respect of the HOMO. This lowering of the LUMO first increases *E*_a_ to a maximum value when both parabolas have the same energy after which *E*_a_ decreases again until the parabolas intersect at their minimum as the system approaches the inverted Marcus region beyond which *E*_a_ remains 0 meV as explained earlier (Section 2.5). Yuan *et al*.^[27]^ experimentally observed a bell‐shaped *E*_a_ *vs. V* curve (Figure 7b) and the data fitted well to the model by Migliore *et al*.^[16b]^ (Section 2.5) demonstrating the transition to activtionless inverted Marcus region at around −0.8 V.

In redox‐active molecular junctions, electron‐vibration interactions cannot be neglected. For instance, Thomas *et al*.^[45]^ observed vibrational excitations in junctions (Figure 7c) based on zinc‐porphyrin compound **12**. As shown in the charge stability diagram (Figure 7d), the equally spaced conductance lines (indicated with arrows) are assigned to vibrational modes of the molecule. To model charge transport in this molecular junction, Thomas *et al*.^[45]^ employed the model by Sowa *et al*.^[16d]^ (Section 2.5), and the excellent agreement between experiment and theory allowed them to identify the vibrational models involved with charge transport. Vibrational excitations have been frequently observed in other studies as well.^[46]^ These examples showcase the complexity of the mechanism of charge transport in redox‐active molecular junctions.

## 4 Functional redox‐active molecular junctions

### 4.1 Molecular diodes

**Figure 8:**
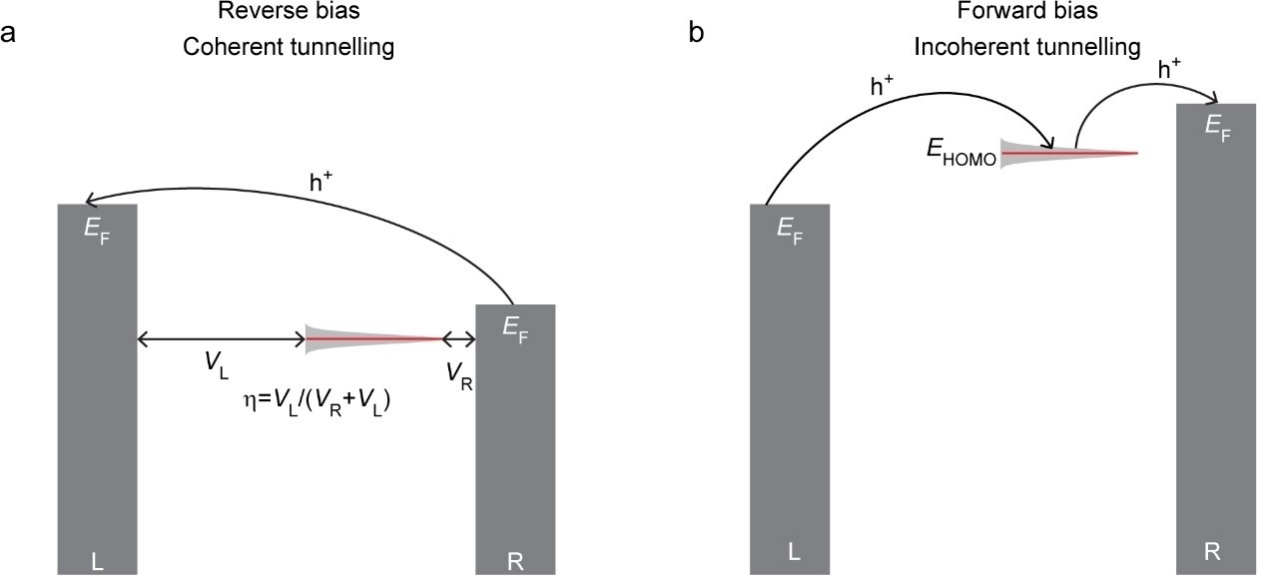
Energy level diagram at reverse bias (a) and forward bias (b) of a molecular diode that changes the mechanism of charge transport between in‐ and coherent tunnelling as function of bias polarity. The red bars indicate the HOMO position and the black arrows depict the mechanism of charge transfer.

Most approaches to develop molecular diodes rely on strong dipoles,^[47]^ D−b−A compounds,[^1c^, ^2a^, ^48^] or asymmetrical molecule‐electrode interfaces operating in the coherent tunnelling regime.^[49]^ In general, these approaches yield molecular diodes with moderate rectification ratios *R* of <10 defined as

(14) $$R=\left|\frac{J_{for}}{J_{rev}}\right|\;$$

where *J*_for_ and *J*_rev_ are the current densities at forward and reverse bias.[^1b^, ^1c^, ^2a^, ^47^, ^48^, ^49^] However, the best performing molecular diodes are based on redox‐active molecules enabling a bias polarity dependent change in the mechanism of charge transport from coherent tunnelling in the off‐state (at reverse bias) to incoherent hopping in the on‐state (at forward bias) as shown in Figure 8. Here the asymmetrical potential drop (*V*_R_ and *V*_L_) along the molecule is essential for rectification which is given by the potential division parameter *η*=*V*_L_/(*V*_L_+*V*_R_). When *η*≈1, the HOMO closely follows the changes in *E*_F,R_ while the opposite is true for *η*≈0 (as indicated in Figure 8), but the junctions do not rectify when *η*≈0.5.[^16e^, ^50^] As indicated in Figure 8, because of the asymmetrical coupling of the molecular orbital (in this case the HOMO) to the electrodes, only at forward bias the HOMO falls in the conduction window resulting in a change in the mechanism of charge transport.

**Figure 9:**
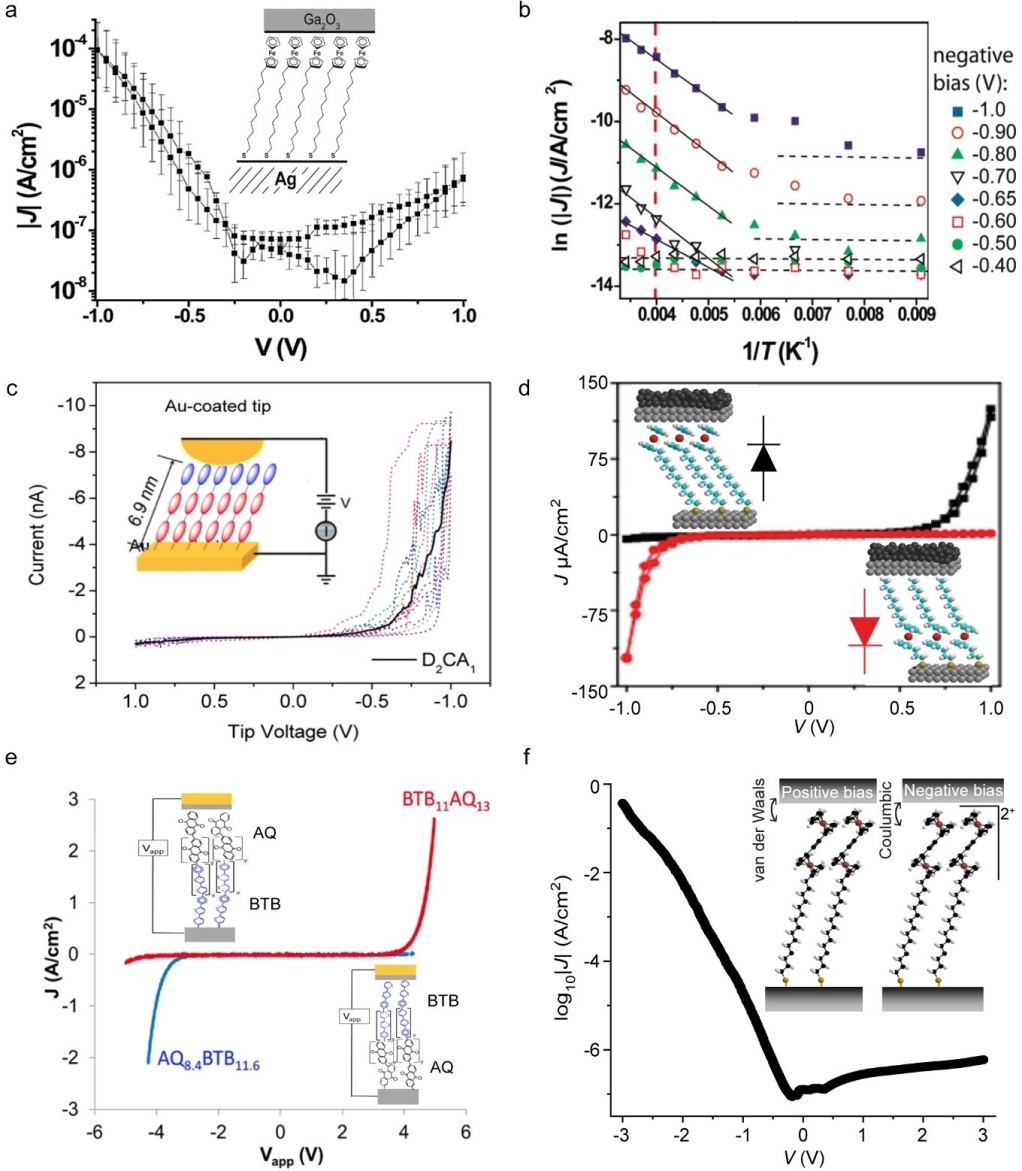
(a) Log‐average *J*(V) curve of compound **13** based large‐area molecular junction and (b) the corresponding Arrhenius plots for different potentials at negative bias. Reprinted with permission from ref 51, copyright 2010 American Chemical Society. (c) Averaged *I*(V) curve (black solid) for 15 traces (coloured dashed lines) collected in an AFM‐based junction with compound **14**. Reprinted with permission from ref 53, copyright 2014 American Chemical Society. (d) *J*(V) curves of large‐area molecular junctions with Fc in different positions (compound **15**) demonstrating the reversal of rectification. Reprinted with permission from refs 50 and 54, copyright 2015 Nature Springer. (e) *J*(V) curves recorded from a large‐area junction with BTB/AQ (red solid line) and AQ/BTB reversed (compounds **16** and **17**, blue solid line) junctions. Reprinted with permission from ref 55, copyright 2016 American Chemical Society. (f) Semi log plot of the *J*(V) curve of large‐area junction with compound **18**, the inset shows that Γ changes due to a change in the molecule‐electrode interaction from a van der Waals to an electrostatic interaction. Reprinted with permission from ref 2c, copyright 2017 Nature Springer.

We have obtained *R* >100 at ±1.0 V for junctions with Fc‐alkanethiolates (Figure 9a, compound **13**) where the redox active Fc units (at which the HOMO is centered) are in close contact with the right electrode but separated from the left electrode by the long alkyl chain resulting in *η*≈1.^[51]^ Temperature dependent measurements (Figure 9b) show that at negative bias hopping dominates the mechanism of charge transport (*E*_a_=77 meV) while at positive bias the mechanism of charge transport is activationless.^[51b]^ Based on a similar design, Yuan *et al*.^[52]^ increased *R* to 1000 at ±1.0 V. A similar change in the mechanism of charge transport has been also reported by Luo *et al*.^[53]^ for a molecular diode based on the redox‐active D−b−A compound **14** with *R* of 30 at ±1.0 V (Figure 9c).

The direction of rectification can be controlled by controlling the relative position of molecular frontier orbitals with respect to the electrodes which in effect changes *η*≈1 to *η*≈0 (or vice versa) which was first demonstrated in Fc (compound **15**) based molecular diodes shown in Figure 9d.^[50]^ Bayat *et al*.^[55]^ demonstrated reversal of rectification of a molecular diode by changing the order of the donor and acceptor units inside the junction as shown in Figure 9e. They used diodes based on bisthienylbenzene (BTB, compound **16**) as the donor and anthraquinone (AQ, compound **17**) as the acceptor. Chen *et al*.^[2c]^ reported *R*=6.3×10^5^ at ±3 V (Figure 9f) which is comparable with that of conventional diodes based on semiconductors. The molecular diode is based on an asymmetrically coupled Fc−C≡C−Fc redox unit **18** following a similar mechanism as depicted in Figure 8. This large rectification ratio was realized because of charging of the Fc units increases the molecule‐electrode coupling strength with the electrodes (*i. e*., Γ) in only one bias direction (Figure 9f inset) improving *R* by 2–3 orders of magnitude compared to other Fc‐based diodes.

### 4.2 Molecular switches

**Figure 10:**
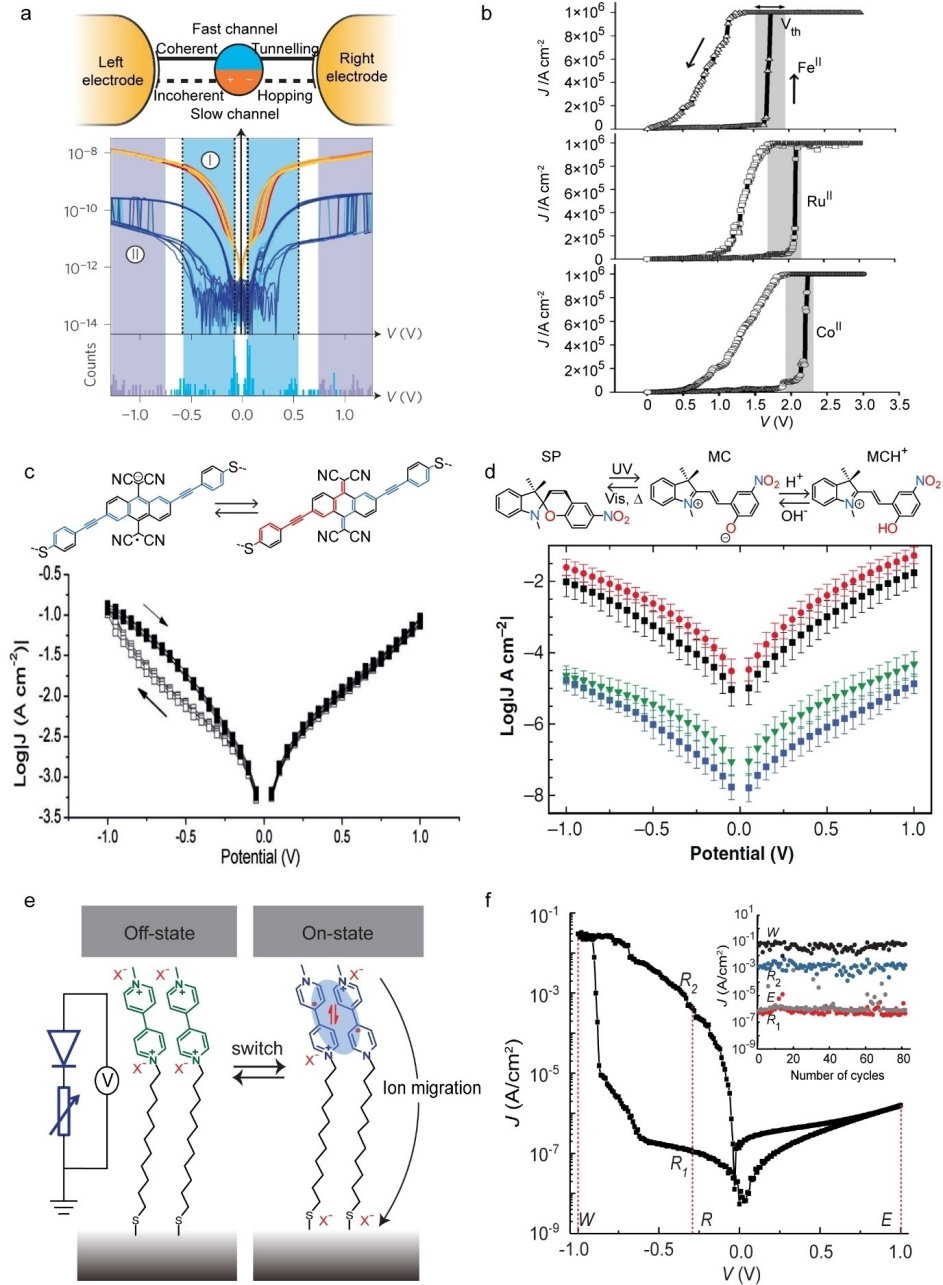
(a) Single‐molecule junction with compound **19** along with 50 representative *I*(V) curves for the junction. Reprinted with permission from ref 3a, copyright 2016 Nature Springer. (b) *J*(V) curves for large‐area molecular junctions with compound **20**. Reprinted with permission from ref 3c, copyright 2012 Wiley‐VCH. (c) Log‐average *J*(V) curves for large‐area junctions with compound **21** and the schematic illustration of the switching between cross‐conjugated quinoid form and reduced linear‐conjugated hydroquinoid form. Reprinted with permission from ref 3d, copyright 2018 Wiley‐VCH. (d) Schematic representation of switching between SP, MC and MCH^+^ and the corresponding log‐average *J*(V) curves in large‐area junctions. Reprinted with permission from ref 56b, copyright 2019 Wiley‐VCH. (e) Schematic illustration of dimerization in compound **24** based large‐area junction. The blue shades indicate dimer formation. The black arrow indicates ion migration. (f) Representative *J*(*V*) curve where *R*_1_ and *R*_2_ represent the low and high conductance states and the corresponding endurance plots. Reprinted permission from ref 3e and ref 58, copyright 2020 Nature Springer and American Institute of Physics.

Molecular switches can toggle between two different conductance states under the action of external stimuli, such as, light, magnetic field, changes in pH or electrical filed.[^1b^, ^1d^, ^1f^, ^1g^, ^3d^, ^56^] Electrical field driven molecular switches are interesting candidates for nonvolatile random‐access memory applications because they do not need external stimuli to switch. In this context, redox‐active molecules are of particular interest because they provide different redox‐states under applied bias enabling to change the conductance state.^[9]^ For example, Schwarz *et al*.^[3a]^ reported conductance switching in break junctions with molecular wires (compound **19**) with a Mo redox centre. The junctions suffered from poor reproducibility and were divided in 2 groups based on their performance: group 1 had current on/off ratios ranging from 1.5 to 20 while group 2 had current on/off ratios of up to 1000 (Figure 10a). They proposed a mechanism of charge transport based on a model developed by Migliore and Nitzan.^[9]^ The molecule provides 2 conduction channels: a fast channel through which the mechanism of charge transport is coherent tunnelling mediated by a delocalized molecular orbital, and a slow channel through which charges hop incoherently mediated by a highly localized molecular orbital on the redox‐centre (Figure 10a). The redox‐state of the molecule determines whether the fast channel (switch is on) or slow channel (switch is off) dominates charge transport. A disadvantage of this approach is that the switching proceeds stochastically. Such stochastic events have been studied by Arielly *et al*.[^11b^, ^57^] and they demonstrated real‐time detection of single redox‐events resulting in telegraph noise in *I*(V) and *I*(t) measurements (with ms resolution, extended to ps resolution by optical means) in Fc‐based molecular junctions at 77 K.

Control over the redox‐state makes it possible to control the switching voltage. For instance, Seo at al.^[3c]^ have demonstrated conductance switching in junctions with a transition metal II (M^II^) bis‐acetylthiophenylterpyridine complex **20** with M=Fe, Ru, Co. The threshold voltage measured over 50 devices for each complex is 1.5–1.9 V for Fe, 1.7–2.1 V for Ru, and 1.9–2.3 V for Co (Figure 10b), which is in good agreement with the δ*E*_ME,L_ value for each complex. Carlotti *et al*.^[3d]^ reported the conductance switching in 11,11,12,12‐tetracyanonaphtho‐1,4‐quinodimethane (TCNAQ, a good electron acceptor, compound **21**) SAMs based junction with a current on/off ratio of 6 which was attributed to the modulation of the bond topology through reduction/oxidation of the TCNAQ molecules (Figure 10c).

Tunnel junctions naturally operate in extremely high electric fields of 0.1–1 GV/m. Therefore, it is challenging to stabilize different redox‐states of the molecules inside the junctions by relying solely on the re‐organization energy *λ* and associated *E*_a_. To avoid spontaneous back switching, additional charge stabilization mechanisms are needed to improve the performance of molecular junctions. Kumar *et al*.^[56b]^ reported a nonvolatile molecular memory device based on the reversible chemical locking of spiropyran (SP, compound **22**; Figure 10d). SP is a well‐known light switch that changes reversibly to the open‐ring zwitteronic merocyanine (MC, compound **23**). However, MC spontaneously thermalizes back to SP. By exposing MC to acid, MCH^+^ is formed which blocks the reverse reaction resulting in devices with stable on‐states (red curve in Figure 10d) and current on/off ratios of 10^3^. Following a very similar approach, Darwish *et al*.^[59]^ demonstrate stable switching via SP↔MC↔MCH^+^ interconversion in single‐molecule junctions. Recently, we^[3e]^ reported a dual‐functional molecular switch based on viologen (Figure 10e, compound **24**) with high current on/off ratios of 6.7×10^3^ and rectification ratios of 2.5×10^4^. This combined diode (D) and variable resistor (R) functionality is called 1D‐1R memory which is promising to reduce power consumption in cross‐bar arrays.^[60]^ In this example, dimerization of the viologen radical cation accompanied by directional migration of the counterions within the junction stabilizes reduced state (on state) of the junction enabling non‐volatile memory. Figure 10f shows the current response of 80 read‐write‐read‐erase cycles.

### 4.3 Comparison between single molecule and large‐area junctions

**Figure 11:**
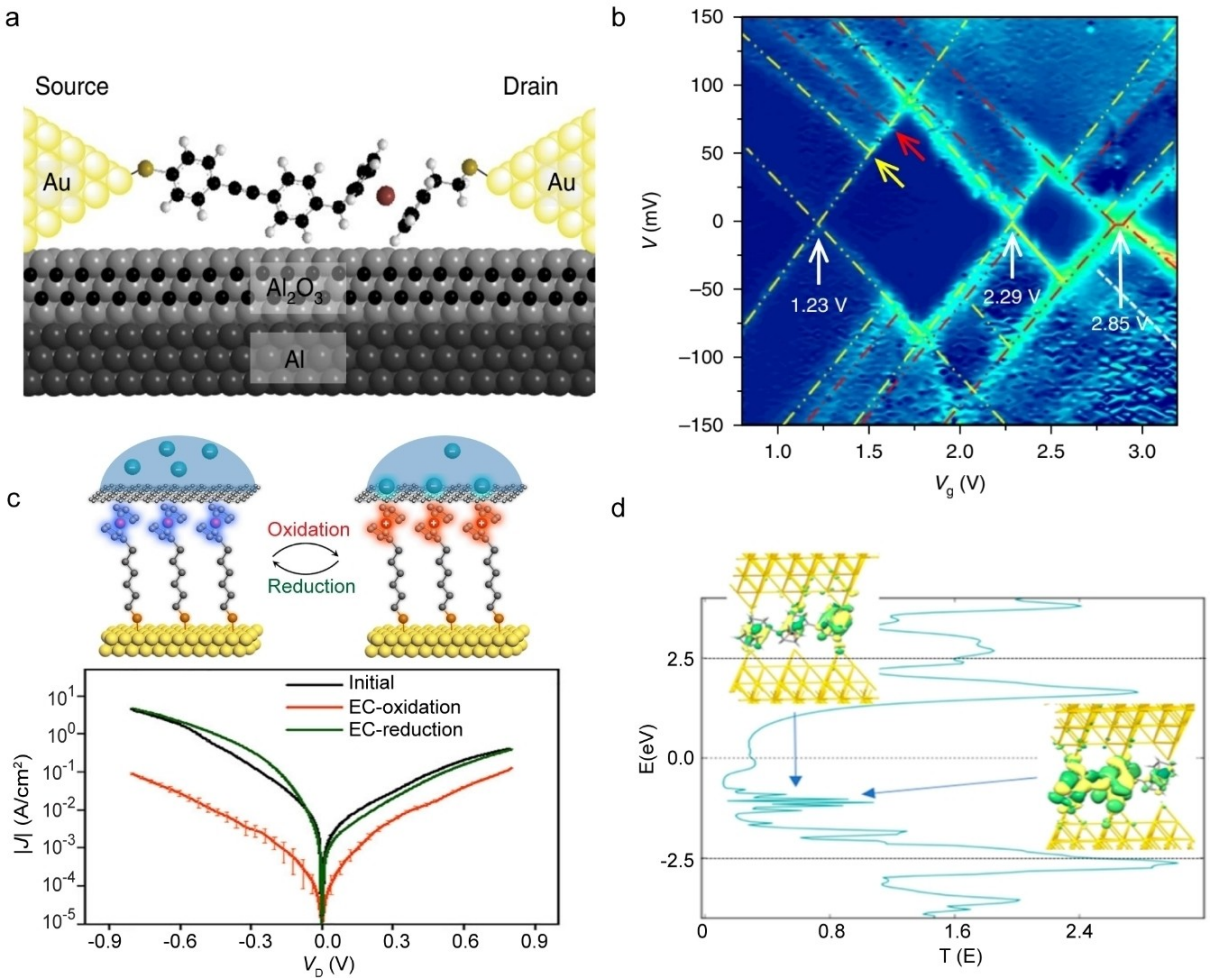
(a) Illustration of the single‐molecule transistor with derivative of compound **11** (n=1) and (b) the corresponding differential conductance stability diagram as a function of bias voltage (*V*) and gate voltage (*V*_g_) at 4 K. Reprinted with permission from ref 27, copyright 2018 Nature Springer. (c) Schematic illustration of the electrochemical oxidation and reduction in a large area junction with a derivative of compound **13** and the current (*J*_D_) ‐voltage (*V*_D_) curves of the junction under different redox‐states. Reprinted with permission from ref 62, copyright 2020 Cell Press. (d) Calculated transmission curve of a single molecule junction with compound **3** with n=3. Reprinted with permission from ref 30, copyright 2019 American Chemical Society.

Generally speaking, single molecule and large‐area molecular junctions are two complementary approaches to study the mechanisms of charge transport at the molecule length scales. For instance, a single molecule junction can be coupled to a third electrode, the gate electrode, to tune the energy level aliment within the molecular junction allowing us to precisely control the redox‐state of the molecule (Figure 1). Figure 11b shows the stability diagram of a single molecule junction with a derivative of compound **11** (Figure 11a) where 3 different redox‐states were accessible within the given bias window as indicated by the Coulomb diamonds.[^27^, ^61^] The Fermi‐levels of the source and drain electrode with respect to the molecular levels were controlled by the gating electrode, resulting in the crossing of the Fermi‐levels with the molecular orbitals as indicated by the white arrows where the oxidation state of the molecule changes. Large area molecular junctions typically lack a gate electrode, but recently a large‐area junction of the form of Au−SAM//graphene/electrolyte with a derivative of compound **13** has been reported where the redox‐state of Fc could be controlled.^[62]^ Here, the oxidation state of the Fc units can be controlled via electrochemical gating through the graphene top electrode. In this device configuration, the rectification of the junction could be turned on and off by controlling the redox state Fc moieties (Figure 11c).

The disadvantage of single molecule junctions over large‐area junctions is that they suffer from large junction‐to‐junction variations due to uncertainties in the atomistic configuration of the junction (*i. e*., the exact molecule‐electrode coupling geometry),[^1a^, ^1d^, ^1e^, ^1g^] which can be quite dynamic at room temperature^[63]^ (and therefore often single molecule experiments are conducted under cryogenic conditions).[^1d^, ^1e^, ^4a^, ^5a^, ^27^, ^64^] Consequently, the analysis of large numbers of data obtained from thousands of junctions is required. In contrast, large area junctions are stable and the monolayer structure supported by the bottom electrode can be independently investigated greatly reducing the junction‐to‐junction variations and the amount of data that is needed (often a few dozens of junctions suffice). For these reasons, large‐area junctions may find first commercial applications in the foreseeable future.^[65]^

Normally, to observe redox‐events in junctions, the charge carrier must reside long enough on the molecule to be detectable at the experimental time‐scales of electrical measurements.[^9^, ^11b^, ^57^] In other words, the junctions should be in the weak coupling regime where the redox‐unit is decoupled from the electrodes (small values of Γ) unless the junctions are probed with elaborate ultra‐fast optical techniques.^[11b]^ Low Γ values, however, results in small currents (Equation 3) which can be challenging to measure in single‐molecule experiments. In contrast, in large‐area junctions, insulating groups such as alkyl chains, can be readily incorporated to ensure weak coupling of the redox‐units with the electrodes, but the overall currents are high enough to be readily measurable (even at low applied voltages close to 0 V) since many molecules are involved in charge transport. In single molecule junctions (at least ideally) atomically sharp electrodes are used, but, in principle, highly unsaturated metal atoms are chemically reactive and interact with the molecules strongly which can result in strong‐molecule electrode coupling switching the mechanism of charge transport to coherent tunnelling (as discussed in Section 3.1). For instance, as shown in Figure 4c, the Au tip can readily interact with the cyclopentadienyl rings of the Fc units forming a new electronic state with a resonance close to Fermi‐level (indicated by the blue arrows in Figure 11d) greatly enhancing the coherent tunnelling rate. Figure 11d shows the calculated transmission curves by DFT for Au−Fc_3_−Au junctions (shown in Figure 4c) and the molecular orbital that corresponds to the low energy resonance.^[30]^ The molecular orbital is highly delocalized over the molecule and both electrodes because the Au tip interacts strongly with the cyclopentadienyl rings of the Fc units. In contrast, in large‐area junctions the top‐electrode is normally deposited using gentle methods to avoid damaging the monolayers during the fabrication process and therefore the monolayer‐top electrode interaction is often weak (van der Waals contact) resulting small Γ values which explains why in large‐area junctions it is relatively straightforward to observe redox‐events.

## 5 Conclusions & Outlook

In summary, redox‐active molecular junctions provide exiting possibilities to control the mechanism of charge transport at the molecular length scales and to induce electronic function. For instance, tunnelling decay coefficients and activation energies for charge hopping can be reduced to a minimum which are promising to realize energy efficient nano‐electronic devices. A challenge is to rationally design molecular devices due to the complex interplay of molecular and supramolecular structure of the junctions, molecule‐electrode interfaces and associated energy level alignment, and changes in the mechanism of charge transport, all of which affect the device performance. By far in most studies molecular junctions are treated as static entities and their properties are modelled using static DFT models.[^1a^, ^1c^, ^1f^, ^1g^, ^3a^, ^3e^, ^25a^, ^30^, ^31^, ^45^] Inherent to redox‐process, the molecules rearrange[^9^, ^15^, ^16b^] and changes in the charge‐state of the molecule(s) result in changes in the potential drops,[^9^, ^16e^] images charge effects,[^1c^, ^1g^, ^13^] or migration of counterions (in cases where they are present[^2b^, ^3e^, ^25b^, ^66^]). Thus, it is important to study the dynamic aspects of molecular junctions in much more detail and also, from a theoretical perspective, to develop models that account for the dynamics of the junctions to capture the switching events of the molecules. To obtain stable and optimal device performance, the molecules should have relatively long‐lived charge states that have to be stabilized in high electric fields present in all molecular junctions. So far only a few approaches to stabilize on/off states have been reported.[^3e^, ^56b^, ^59^] Therefore, new chemical designs of molecules with the capability to stabilize charge on the molecule are needed. Interesting approaches could involve molecular junctions where charge injection is (inspired by biological systems) coupled to proton transfer^[67]^ or intramolecular reversible bond formation^[68]^ to realize stable functional molecular switches inside junctions. Often molecules are studied with mobile counterions that can migrate to the molecule‐electrode interfaces and potentially disturb the SAM packing or adversely change the potential drop profile in the junction, but approaches where the counterions are tethered to the molecules are promising to control ion movement and associated potential drop profiles in junctions. To conclude, redox‐active molecular junctions have shown to generate devices with excellent properties and are very interesting to study the fundamentals of charge transport which could ultimately lead to devices with reduced footprints and power consumption. There is plenty of scope for improvement via chemical engineering and design of the molecular precursors while keeping in the mind the dynamics of the molecules inside the junctions and the molecule‐electrode interfaces in response to changes in the redox‐states of the molecules.

## Conflict of interest

The authors declare no conflict of interest.

## Biographical Information

*Yingmei Han received her B.Sc. degree in chemistry in 2015 from the school of chemistry and chemical engineering, Nanjing University, China. Currently she is a Ph.D. student at department of chemistry, National University of Singapore, under the supervision of Prof. Christian A. Nijhuis. Her research interests include the rational design of functional molecular junctions for applications in molecular memory and rectification, and charge transport mechanisms across these molecular junctions*.

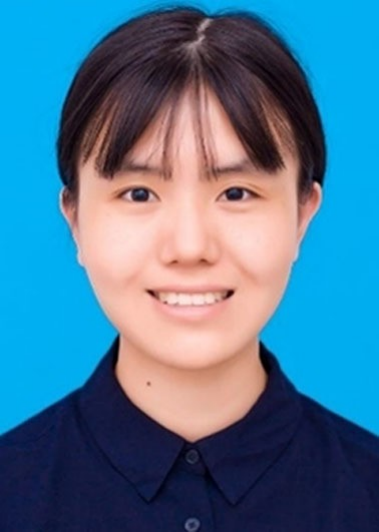

## Biographical Information

*Christian A. Nijhuis is an Assoc. Prof. at the Department of Chemistry at the National University of Singapore (NUS). He obtained his Ph.D. degree from the University of Twente in 2006 (Cum Laude; top 5 %) under the supervision of Prof. David N. Reinhoudt and Prof. Jurriaan Huskens and then started (2007) a post‐doctoral fellowship in the group of Professor George M. Whitesides. After receiving the National research foundation (NRF) research fellowship of Singapore in 2010, he started his group at NUS. His current research interests include molecular electronics, quantum plasmonics, nanofabrication, self‐assembly and surface science*.